# Increased Sialylation of Anti-Thomsen-Friedenreich Antigen (CD176) Antibodies in Patients with Gastric Cancer: A Diagnostic and Prognostic Potential

**DOI:** 10.1155/2014/830847

**Published:** 2014-09-04

**Authors:** Oleg Kurtenkov, Jelena Izotova, Kersti Klaamas, Boris Sergeyev

**Affiliations:** Department of Oncology and Immunology, National Institute for Health Development, Hiiu 42, Tallinn 11619, Estonia

## Abstract

*Aim*. To study whether alterations in the sialylation of antibodies (Ab) specific to the Thomsen-Friedenreich (TF) glycotope have a diagnostic and prognostic potential in gastric cancer. *Methods*. Serum samples were taken from patients with gastric carcinoma (*n* = 142) and controls (*n* = 61). The level of TF-specific antibodies and their sialylation was detected using ELISA with synthetic TF-polyacrylamide conjugate as antigen and sialic acid-specific *Sambucus nigra agglutinin* (SNA). *Results*. The level of TF-specific IgM was significantly decreased in cancer compared with controls (*P* ≤ 0.001). Cancer patients showed a higher level of SNA binding to anti-TF IgM and IgA (*P* ≤ 0.001) irrespective of disease stage, tumor morphology, and gender. Changes in the SNA/Ab index demonstrated moderate sensitivity (66–71%) and specificity (60–73%) for stomach cancer. The best diagnostic accuracy (100%) was achieved in 29% patients with high SNA binding and low anti-TF IgM level. This subset of patients demonstrated the poorest survival. *Conclusion*. Our findings are the first evidence that the increased sialylation of TF-specific Abs combined with a low level of anti-TF IgM is strongly linked to gastric cancer and patients survival, which can be used as a novel biomarker for cancer detection and prognosis.

## 1. Introduction

Early detection is vital for an effective treatment of cancer. The discovery and characterization of new easily applied sensitive and specific cancer biomarkers are promising ways for further success in early cancer diagnostics, patient monitoring, and prognostics. The altered glycosylation observed in cancer cells leads to the expression of modified tumor-associated glycans (TAG) such as Thomsen-Friedenreich antigen (Gal*β*1-3GalNAcα/*β*-O-Ser/Thr; TF, CD176) that may be autoimmunogenic and may be recognized by autoantibodies [1–6]. TAG are considered as promising targets for cancer immunotherapy [6–8]. The TF glycotope overexpression observed in the majority of adenocarcinomas and the reduced level of anti-TF antibodies are associated with more aggressive tumors and the induction of invasion, cancer surveillance mechanisms, and patients survival rate [3, 7, 9–13]. The TF antigen seems to play a crucial role in the adhesion of cancer cells to the endothelium through the interaction with galectin-3, thereby promoting metastases [14, 15]. In cancer patients, an abnormal glycosylation pattern has been demonstrated for many circulating glycoconjugates [2, 4, 16–18], including immunoglobulins which have a set of glycoforms differing in number, type, and site of oligosaccharide attachment [19]. It is now clear that the N-glycans of the Fc-fragment strongly influence IgG-Fc*γ* receptor interactions and thus the Fc-mediated effector mechanisms [20–22].

Appreciable amounts of TF-specific antibodies of different isotypes are present in normal human serum. Their level is decreased in patients with cancer although there are large interindividual variations [1, 3]. Little attention has been paid so far to the glycosylation of naturally-occurring TF-specific antibodies. Recently, we reported that the sialic acid specific SNA lectin reactivity of anti-TF IgG determined in the total IgG purified from the serum of patients with stomach cancer was significantly decreased compared to that of healthy blood donors and patients with nonmalignant gastric diseases [23]. As detected by LC-ESI-MS, the sialylation of the total_IgG Fc glycan was also found to be much less pronounced in cancer patients [24]. These findings prompted us to further investigate whether the sialylation of anti-TF Abs of various isotypes reveals cancer-associated changes that could be used as a biomarker of gastric cancer. The* Sambucus nigra* agglutinin (SNA) directed against glycans with the terminal *α*2,6-linked sialic acid has been shown to bind mostly to Fab glycans that, in contrast to Fc glycans, are fully sialylated and strongly SNA reactive [25–28].

In the present study, we show that, in contrast to the anti-TF IgG or Fc glycans detected in the purified total IgG samples, the sialylation of TF-specific IgM and IgA antibodies is significantly increased in patients with gastric cancer already in the early stages of the disease. The combined analysis of the anti-TF IgM antibody level and SNA reactivity revealed its promising diagnostic (ACC = 69%) and prognostic potential. Moreover, using the further stratification of patients by these two parameters, we were able to diagnose gastric cancer in 29% of patients with 100% accuracy, irrespective of cancer stage, tumor morphology, or gender. In addition, the poor survival of cancer patients with a low level of TF-IgM and high SNA reactivity of TF-specific antibodies was demonstrated.

## 2. Material and Methods

### 2.1. Subjects

Serum samples were taken from healthy blood donors, patients with benign stomach diseases, and individuals with histologically verified gastric carcinoma (Table 1). The investigation was carried out in accordance with the ICH GCP Standards and approved by the Tallinn Medical Research Ethics Committee, Estonia. A written informed consent was obtained from each subject. Tumor staging and morphology were based on the histopathological (pTNM) classification of malignant tumors and evaluated according to the system of Lauren 1965 [29] as an intestinal or diffuse type of tumor growth. The serum samples were stored in aliquots at −20°C until use.

### 2.2. The TF-Specific Antibody Assay

The level of anti-TF IgG, IgM, and IgA was determined by the enzyme-linked immunosorbent assay (ELISA) as described elsewhere [12], with minor modifications. Briefly, the plates (Maxisorp, Nunc, Roskilde, Denmark) were coated with a synthetic TF-polyacrylamide conjugate (TF-PAA, Lectinity, Russia, 10 mol% of carbohydrate) in the carbonate buffer, pH 9.6. After the overnight incubation, triple washing and blocking with a Superblock solution (Pierce, USA) for 15 min at 25°C the serum samples diluted 1 : 50 in PBS-0.05% Tween (Tw) were applied for 1.5 h at 25°C. After the subsequent washing with PBS-Tw, the level of bound anti-TF Ab was determined using the alkaline phosphatase conjugated goat anti-human IgG, IgM (Sigma, USA), or IgA (Dako, USA) and p-nitrophenylphosphate disodium hexahydrate (Sigma, USA). The absorbance values were read at 492 nm (Tecan Reader, Austria). The optical density value (O.D.) of control wells (blank: the Superblock solution instead of TF-PAA) was subtracted from that of Ab-coated wells and each sample was analysed in duplicate.

### 2.3. The SNA Lectin Reactivity of TF-Specific Antibodies

The lectin reactivity of TF glycotope specific antibodies was measured in a similar way, except that the binding of the neuraminic acid (sialic acid) specific* Sambucus nigra* agglutinin (SNA) to the absorbed anti-TF antibodies was determined as described by Kodar et al. [23]. The biotinylated SNA (Vector Laboratories Inc., USA) in 10 mmol/L Hepes, 0.15 mol/L NaCl, 0.1 mmol/L CaCl_2_, and pH 7.5 was applied at a concentration of 5 *μ*g/mL for 1.5 h at 25°C. The bound lectin was detected with a streptavidin-alkaline phosphatase conjugate (Dako, USA) and p-nitrophenylphosphate (Sigma, USA). The optical density value (O.D.) of control wells (no sample) was subtracted from that of Ab-coated wells to determine the lectin binding. Each sample was analysed in duplicate. The value of the SNA binding to all TF-specific Abs and the ratio of SNA binding to TF-specific IgG, IgM, and IgA level (SNA/Ig index) were determined.

### 2.4. Statistical Analysis

Comparisons between the groups were made using the nonparametric Mann-Whitney *U* test for unpaired data (or Student's *t*-test, where appropriate), the discriminant analysis, and the Pearson two-tailed correlation. The survival of cancer patients with weak and strong response was analysed by the Kaplan-Meier method, using the Estonian Cancer Registry database. The median of anti-TF antibody or SNA binding levels was used as cut-off. Patients whose median is equal to or greater than the corresponding median O.D. value were classified as strong responders and those with levels below the median as weak responders. The receiver operator characteristic (ROC) curve analysis was used to evaluate the sensitivity and specificity of changes found for stomach cancer. The area under the ROC curve and the *P* value of the ROC curve were calculated. The difference between the groups was considered to be significant when *P* ≤ 0.05. All calculations were performed using the GraphPad Prism 5 and SPSS 15.0 software.

## 3. Results

### 3.1. The Level of TF-Specific Antibodies in the Serum of Cancer Patients and Controls

**Figure 1:**
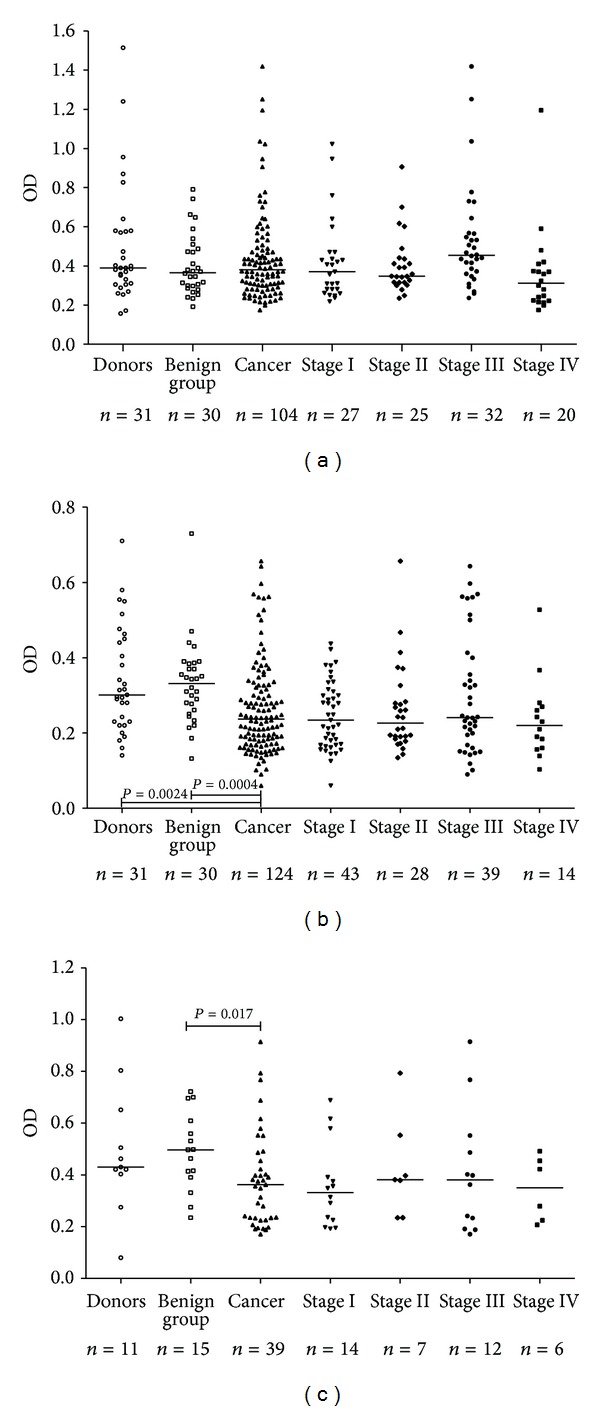
The TF-specific antibody level in patients with stomach cancer and controls. Anti-TF antibody level pattern in controls and cancer patients by stage of cancer; each dot represents one individual and group median is indicated by horizontal lines: (a) anti-TF IgG; (b) anti-TF IgM; (c) anti-TF IgA. *P* values were calculated by the Mann-Whitney *U*test and are shown for significant differences.

**Figure 2:**
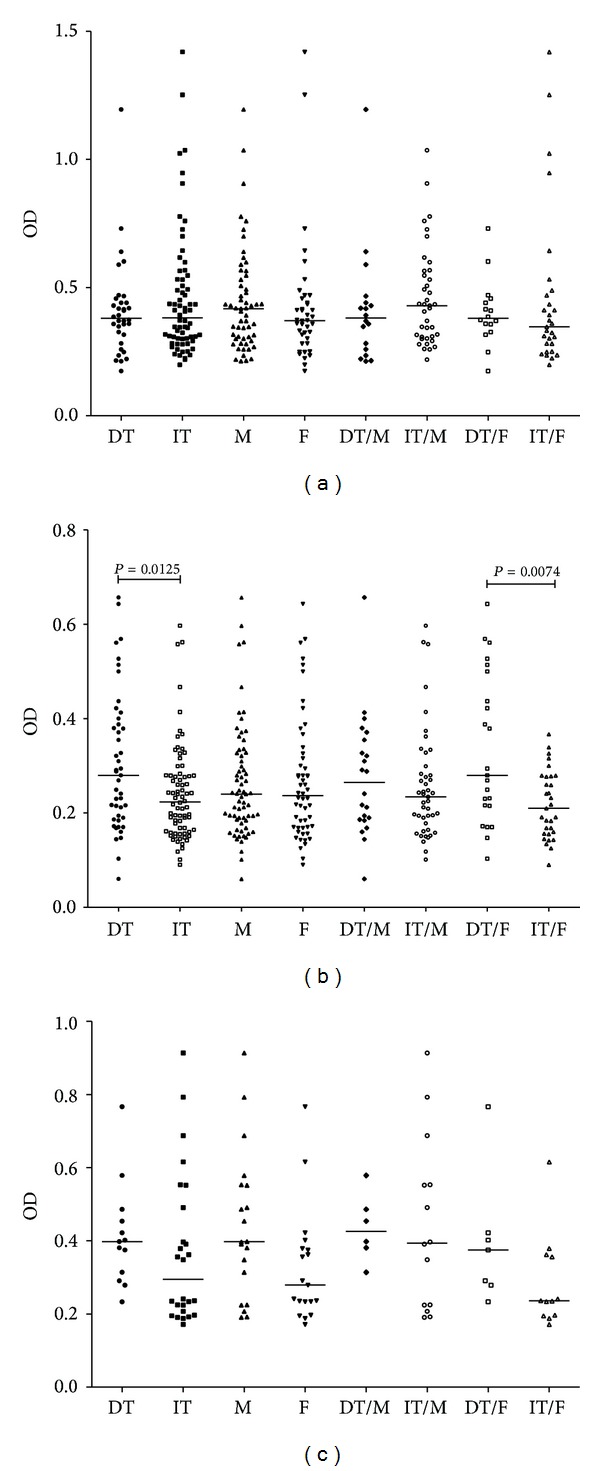
The TF-specific antibody level in cancer patients by gender and tumor morphology. Each dot represents one individual and group median is indicated by horizontal lines: (a) anti-TF IgG; (b) anti-TF IgM; (c) anti-TF IgA. Tumor morphology was evaluated by the Lauren classification as an intestinal (IT) or diffuse (DT) type of tumor growth. M: males; F: females. *P*values are shown for significant differences.

There was no significant difference in anti-TF IgG antibody level between cancer patients and both of the control groups (Figure 1(a)). A trend to a lower IgG Ab level was observed only in stage 4 patients: *P* was 0.033 and 0.09 compared to donors and the benign gastric diseases group, respectively. The anti-TF-IgM serum level was significantly lower in cancer patients than in blood donors (*P* = 0.0024) and the benign diseases group (*P* = 0.0004) and for the combined group of controls (*P* = 0.0001), with no relation to the stage of cancer (Figure 1(b)). This decrease was mostly observed in patients with an intestinal type of cancer (*P* = 0.012), unlike those with a diffuse type of tumor growth, especially in females (*P* = 0.007) (Figure 2(b)). Similar anti-TF IgM Ab levels were observed in blood donors and the benign diseases group (*P* = 0.88). The TF-specific IgA antibody level was also lower in cancer patients than in donors (*P* = 0.06) and the benign diseases group (*P* = 0.017) (Figure 1(c)). Like anti-TF IgM, a lower anti-TF IgA Ab levels were found in patients with intestinal type tumors (Figure 2(c)). For all the groups under study, there were rather big interindividual variations in any Ig isotype. No significant correlations between the levels of anti-TF antibodies of different Ig isotypes were observed in both patients and controls: IgG versus IgM, *r* = −0.1 and IgG or IgM versus IgA, *r* = 0.23–0.31 (*P* > 0.05).

Thus, the TF-specific IgM and IgA antibody levels were decreased in gastric cancer patients irrespective of the stage of cancer with some dependency on tumor morphology, while the anti-TFIgG level was slightly decreased in patients with advanced cancer only.

### 3.2. Interaction of TF-Specific Antibodies with* Sambucus nigra *Agglutinin (SNA)

**Figure 3:**
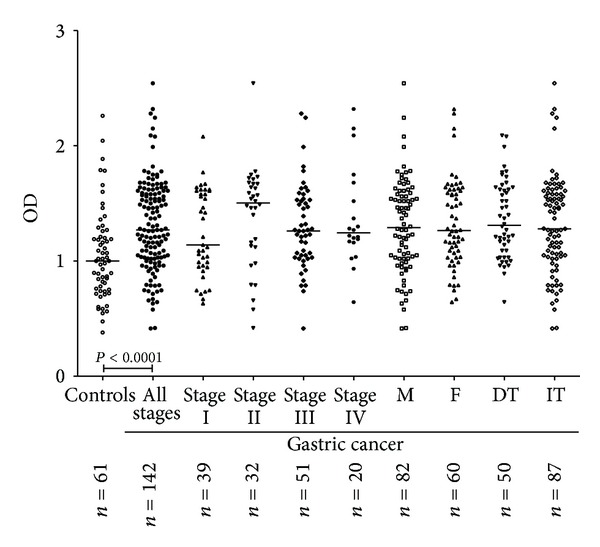
The binding of* Sambucus nigra* agglutinin to TF-specific antibodies in controls and gastric cancer patients by stage of disease, tumor morphology, and gender. DT: Diffuse type of tumor growth; IT: intestinal type of tumor growth. M: males; F: females. *P* values are shown for significant differences.

The binding of SNA to anti-TF Abs (pool of all Ig isotypes) was significantly higher in cancer patients compared with that of blood donors and patients with nonmalignant gastric diseases or the combined group of controls: *P* was 0.0003, 0.005, and <0.0001, respectively, (Figure 3). The increase in the SNA lectin reactivity was not dependent on the stage of cancer except the slightly higher values in stage 2 versus stages 3 or 4 patients (*P* = 0.15) and tumor morphology (DT/IT); it was similar in males and females and in patients under and over 50 (data not shown).

**Figure 4:**
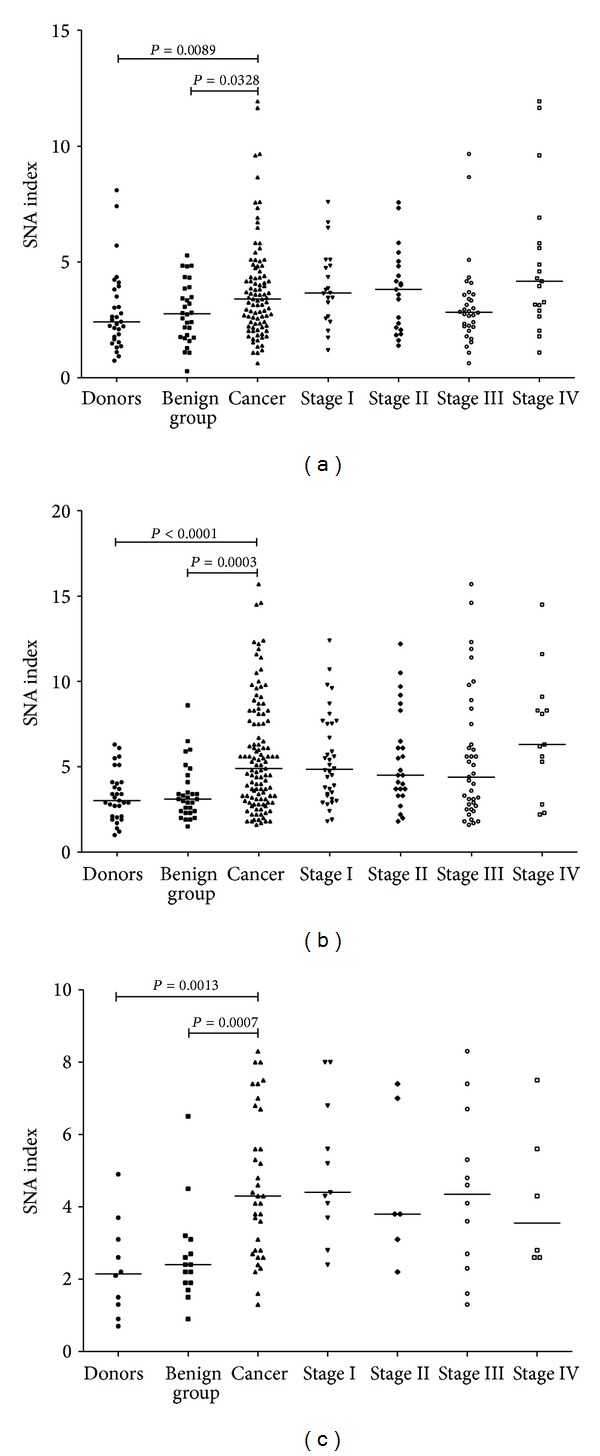
The binding of SNA to anti-TF antibodies depending on the level of TF-specific IgG, IgM, and IgA (SNA/Ig index). (a) SNA/anti-TF IgG, (b) SNA/anti-TF IgM, and (c) SNA/anti-TF IgA index.*P* values are shown for significant differences.

The SNA binding assay results were further correlated with the level of anti-TF IgG, IgM, and IgA, and the SNA binding/Ig level ratio, or the SNA index, was calculated for each Ig isotype. A significant increase of the SNA/anti-TF IgM index was found in patients with cancer unlike both blood donors and the benign diseases group or the combined group of controls: *P* was 0.0001, 0.0003, and <0.0001, respectively (Figure 4). The same was true for anti-TF IgA: *P* was 0.0013 and 0.0007 for donors and the benign diseases group, respectively. An increase of the anti-TF IgG SNA index in cancer was a bit less expressed: *P* was 0.0089 and 0.033 for blood donors and the benign diseases group, respectively.

The discriminant analysis of three variables (level of anti-TF IgG, anti-TF IgM, and SNA binding value as a dependent variable (*n* = 104)) showed that the anti-TF IgM antibodies played the main role in the SNA binding (*P* = 0.0001), while the role of TF IgG was negligible (*P* = 0.82). The anti-TF IgA level was not included in the analysis due to the small number of patients tested.

Thus, the significantly higher SNA reactivity of TF-specific Abs, which reflects the interaction with the terminal alpha 2,6-linked sialic acid of Ab glycans, was revealed in patients with gastric cancer irrespective of disease stage, tumor morphology, or gender. It appears that anti-TF IgM is the main target of the changes observed.

### 3.3. Changes of the Anti-TF Abs Sialylation as Biomarker for Gastric Cancer Diagnostics: Receiver Operator Curve (ROC) Analysis

The sensitivity and specificity of changes of the anti-TF Abs level and SNA reactivity for gastric cancer were assessed using the ROC curves analysis (Table 2).

**Figure 5:**
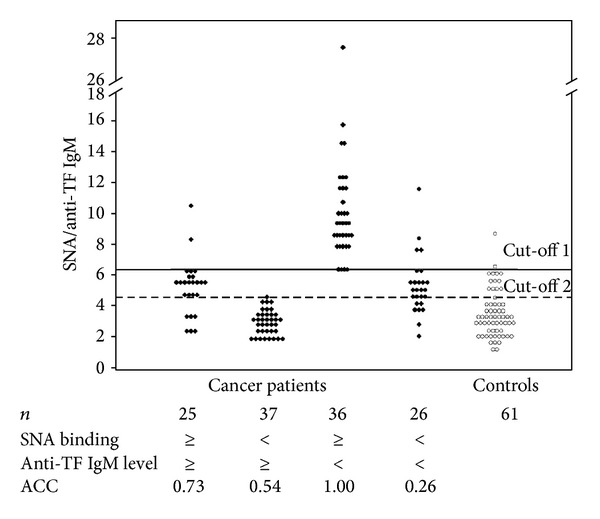
The accuracy of gastric cancer diagnostics (ACC) after the stratification of gastric cancer patients by SNA binding and anti-TF IgM level. The median of the SNA binding and anti-TF IgM level was used to distinguish four subgroups as shown in Figure 5. A solid horizontal line shows the SNA/anti-TF IgM index cut-off 1 (6.1) that allows the best discrimination between subgroup 3 (SNA ≥ IgM <) and controls as determined by the ROC curves analysis. Cut-off 2 (a dotted line) indicates the best discrimination between the combined group of cancer patients and controls. ACC: accuracy of diagnostics as calculated by the ROC curve analysis.

The level of anti-TF IgG showed very low sensitivity, specificity, and accuracy (ACC = 0.56) for gastric cancer. The level of anti-TF IgM demonstrated a bit higher predicted group membership for cancer (ACC = 0.67). Compared to the SNA binding assay alone, a higher diagnostic accuracy was demonstrated for the SNA/IgM and SNA/IgA indexes (ACC = 0.69 and 0.72). Considering that both the anti-TF IgM level and SNA binding values were significantly changed in cancer patients and the respective changes showed quite an opposite direction, the same value of the SNA/anti-IgM index may be obtained if both variables are similarly either low or high. Therefore, cancer patients were further stratified into four subgroups by using the median of SNA binding and anti-TF IgM level values for the combined group of cancer patients as follows: (1) patients with an SNA binding value that is equal to or more than median and anti-TF IgM level that is equal to or more than median; that is, SNA ≥ IgM ≥, (2) SNA < IgM ≥, (3) SNA ≥ IgM <, and (4) SNA < IgM < subgroup (see the table in Figure 5). The controls (the combined group of donors and the benign diseases group) were stratified in a similar way by using the corresponding medians of the cancer group. All subgroups of cancer patients were subjected to the ROC curve analysis for sensitivity, specificity, and accuracy of cancer diagnostics and the results were compared with those of the corresponding subgroup of controls (Figure 5).

It is obvious from Figure 5 that the 3rd subgroup, that is, subjects with a high SNA/anti-IgM ratio due to the high level of SNA binding and low level of TF IgM, exclusively belongs to the cancer patients group. Using the ROC analysis the cut-off level of the SNA/anti-IgM index that allows the best discrimination of cancer patients with high SNA binding and low anti-TF IgM level from controls was determined to be equal to 6.1 (cut-off 1). Only 2 out of 61 control subjects had the SNA/IgM ratio value above this cut-off: one from (≥≥) subgroup and one from (≪) subgroup. No controls belonged to the SNA ≥ IgM < subgroup. It is notable that no appreciable differences between the subgroups in the distribution of patients by stage of disease, tumor morphology, and gender were found (Table 3). Thus, using this approach subgroup 3 (SNA ≥ IgM <) may be selected (36 patients out of 124 (29.03%)) whose specificity, sensitivity, and ACC × 100 of gastric cancer diagnostics have reached 100% (Figure 5). The analogous analysis of patients and controls by SNA binding and anti-TF IgA level, as has been done for SNA and IgM, showed that subgroup 3 (SNA ≥ IgA <) demonstrated the ACC value equal to 52%; that is, there was no discrimination between patients and controls.

### 3.4. Survival Analysis

**Figure 6:**
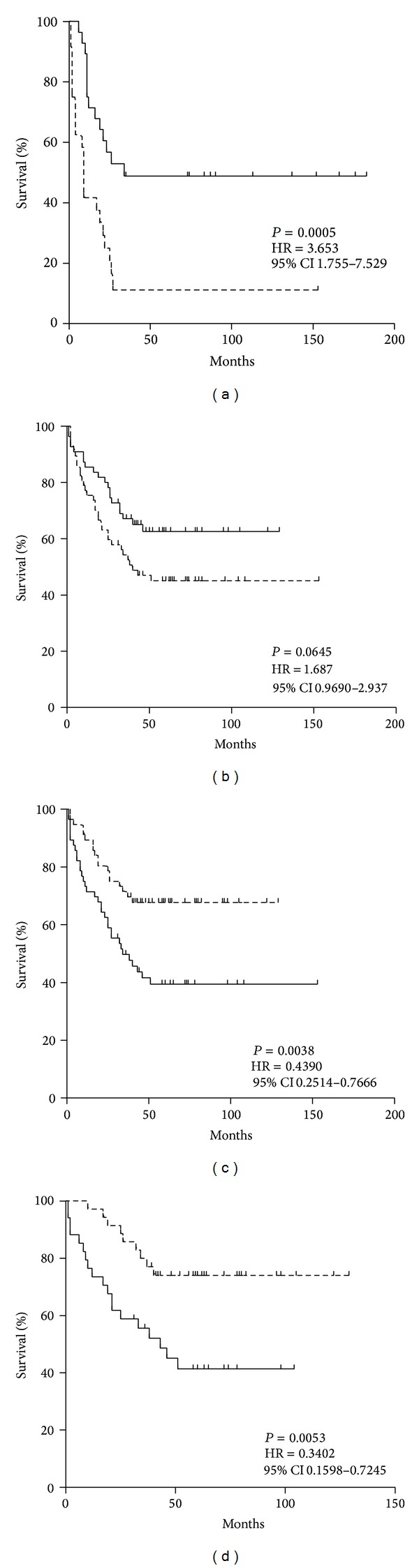
The probability of survival of stomach cancer patients in relation to the level of TF-specific antibodies and their SNA reactivity. Patients with either lower, equal (a dashed line), or higher values (level of Abs or SNA binding) than median (a solid line) are compared using the Kaplan-Meier method. HR: hazard ratio with 95% confidence interval and *P* values are shown. (a) Anti-TF IgG level and survival of patients in stages 3-4 of the disease. (b) Anti-TF IgM level and survival of cancer patients (stages 1–4). (c) SNA/anti-TF IgM index (all patients). (d) SNA/anti-TF IgM index (patients with intestinal tumors).

**Figure 7:**
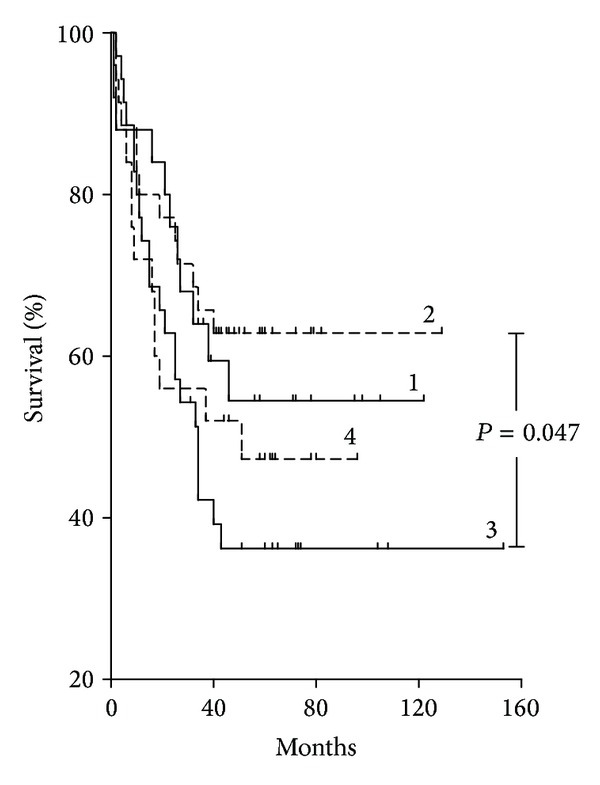
The probability of survival of gastric cancer patients in subgroups stratified by TF-specific antibody SNA reactivity and TF IgM level. The stratification of cancer patients into four subgroups was performed as shown in Figure 5 by using the median of SNA binding and anti-TF IgM level value as cut-off: (1) patients with an SNA binding value that is equal to or more than median and anti-TF IgM level that is equal to or more than median; that is, SNA ≥ IgM ≥, (2) SNA < IgM ≥, (3) SNA ≥ IgM<, and (4) SNA < IgM < subgroup.

The relation of cancer-associated changes in the level of anti-TF antibodies and their SNA reactivity to survival is illustrated in Figures 6 and 7. High level of anti-TF IgG was associated with a better survival in patients of stages 3-4, compared with low responders (*P* = 0.005) (Figure 6(a)). A similar trend was observed for anti-TF IgM level (*P* = 0.064) especially in stage 3 patients (*P* = 0.01) (Figure 6(b)) irrespective of tumor morphology and gender (data not shown). The SNA reactivity of TF-specific Abs (pool of all isotypes) showed no relation to survival (HR = 0.88 (0.53–1.46), *P* = 0.63, *n* = 136). The high SNA binding/anti-TF IgG index did not show any association with survival either (HR = 0.97). In contrast, the high SNA/anti-TF IgM index was associated with poor prognosis (*n* = 112, HR = 0.44 (0.25–0.77), *P* = 0.0038) (Figure 6(c)), especially in patients with intestinal tumors (Figure 6(d)) exhibiting a more pronounced association (*P* = 0.005; HR = −0.34 (0.16–0.72); *n* = 69) in both males and females: *P* was 0.07 and 0.026, respectively. Patients with low differentiated (diffuse type) tumors showed a weaker association (HR = 0.69 (0.29–1.61), *P* = 0.39).

However, in subgroups of patients stratified by SNA reactivity and anti-TF IgM level (as shown in Figure 5 and Table 3) association with survival was different (Figure 7). For instance, group 3 with the highest SNA/anti-IgM index values showed a significantly poorer survival: HR = 0.50 (0.26–0.99), *P* = 0.047 compared with group 2 [<≥] in spite of the highly similar distribution by stage of disease, tumor morphology type, and gender in both groups (Table 3).

## 4. Discussion

The majority of natural tumor-specific Abs belong to germ-line coded IgM antibodies directed mostly against carbohydrate epitopes and may be responsible for Abs-mediated tumour defence [4, 30, 31]. The origin of these Abs is still a matter of debate, but it appears that some of them (anti-TF and anti-alpha Gal Abs of different isotypes) are directed against microbial glycans or antigens cross-reactive with them [32–34]. Since the 1980s [1] it has been established that the level of anti-TF IgM is lower in cancer patients and is related to higher breast cancer risk. It is notable that the level of Abs to many TAGs is often decreased in cancer patients [3, 4, 12]. Circulating autoantibodies to tumor-associated antigens, including TAGs, are considered as promising biomarkers for an early detection of cancer [6, 35] although up to now these antibodies have not shown any sufficient diagnostic accuracy or clinical applicability.

In the present study, we found that all isotypes of TF-specific Abs demonstrated a common trend to a lower level in patients with gastric cancer though IgM Abs revealed the most pronounced decrease. To our knowledge, the level of anti-TF IgA Abs in patients with cancer has not been studied before. The level of anti-TF IgA was similar to that of IgG, but no correlation between anti-TF IgA and the two other isotypes was observed. Rather big interindividual variations in TF-specific Ab level were observed in patients and controls suggesting that some ohter reasons but cancer may be involved, for instance, the profile of individual microbiota.

While many serum glycoproteins exhibit carbohydrate changes in malignancy [5, 17, 18, 36], comparably little is known about the glycosylation of immunoglobulins in patients with cancer, especially in regard to antibodies directed against tumor-associated antigens. Gerçel-Taylor et al. [37] reported that the tumor-derived IgG exhibited more pronounced changes in the glycosylation than serum IgG in patients with ovarian cancer. These authors have supposed that the aberrantly glycosylated serum IgG either may be of tumor origin or may accumulate in tumor tissue. Oaks et al. [38] demonstrated that IgG antibodies against melanoma-associated antigens were much more sialylated compared to the total serum IgG or anti-infectious Abs obtained in melanoma patients, as measured by the SNA lectin binding assay. In some autoimmune disorders, the variable region glycosylation of antigen-specific autoantibodies was also different from that of total IgG, [28]. Thus the determination of the total serum Abs glycosylation does not reflect the glycosylation profile of antigen-specific Abs. This implies that the glycosylation pattern of Abs against the target antigens involved in the pathogenesis of a specific disease may be more informative.

The lectin-based analysis of TF-specific IgG revealed significant alterations in sialo- and fucose-specific lectin reactivity in cancer patients [23]. Interestingly, in patients with gastric cancer the TF-specific IgG antibodies in the total purified IgG were, on the contrary, significantly less SNA-reactive [23] and similar decrease in the purified IgG Fc glycan sialylation was demonstrated by mass spectrometry [24]. Recently, we established a much higher level of anti-TF IgG in purified IgG than in serum samples (unpublished). This suggests that some additional, the so-called “hidden,” anti-TF IgG have been detected in the purified IgG, which may be due to several reasons such as: (i) presence of IgG Abs in a bound form in serum (in complexes with TF-positive ligands); (ii) modification of IgG during the purification (acid elution, dissociation of IC); and (ii) appearance of IgG polyreactivity in the absence of some serum factors that block or inhibit the TF-specific IgG reactivity. Those hidden Abs cannot be detected by conventional methods and may be aberrantly glycosylated (hypersialylated). We suggest that unmasking such hidden Abs and analysis of the whole spectrum (free and hidden Abs) of anti-TF antibodies may lead to the discovery of new biomarkers in tumor immunity and autoimmunity.

To our knowledge, no special studies of TF-specific IgM and IgA antibody glycosylation have been performed before. We established a significant increase of the SNA binding (to a pool of anti-TF Abs) and anti-TF IgM SNA index values in patients with gastric cancer, unlike both control groups. In our lectin-ELISA format, the anti-TF Abs bind the TF-PAA conjugate immobilized on plastic. Thus it might be expected that some SNA reactive glycans, especially in the Fab fragment, would be inaccessible or more accessible to lectin after interaction of Abs with TF, as has been shown for the interaction of mannan binding lectin (MBL) after IgM-antigen interaction [39].

This study showed that the level of anti-TF IgG in serum samples did not change much in the cancer group (Figure 1(a)), but the SNA/anti-TF IgG index was significantly higher in cancer patients (Figure 4(b)) suggesting that IgG Fab sialylation may be also related to the increased SNA binding observed in the cancer group. However, the serum IgM is much more glycosylated compared to IgG and more than 80% of IgM complex glycans are terminated in sialic acid [39]. Therefore, we suggested that, despite its lower level in cancer group, the anti-TF IgM is obviously the main component responsible for the increased SNA lectin binding to anti-TF Abs in our model. In fact, the discriminant analysis of SNA binding has indicated that it is anti-TF IgM but not IgG level that was significantly associated with changes in SNA reactivity of TF-specific Abs in cancer patients.

Several reasons might be considered to explain the higher SNA reactivity of anti-TF Abs in cancer patients: (i) in cancer anti-TF IgM is actually more sialylated due to the altered activity of glycosyltransferases in tumor cells and/or in tumor-bearing host; (ii) the Fab glycans of TF-specific Abs are more accessible to SNA due to IgM conformational modifications after interaction with some ligands, such as MBL or other endogeneous lectins; (iii) the anti-TF IgM sialic acids of controls are masked by some TF-positive ligands that are absent in cancer patients but present in healthy state.

Both the level of anti-TF Abs and SNA binding alone had a relatively moderate diagnostic value in gastric cancer with maximal diagnostic accuracy for anti-TF IgM and IgA level (ACC was 0.67 and 0.72, resp., Table 2). The same was true for the SNA/IgM and SNA/IgG indexes (ACC was 0.69 and 0.64, resp.). However, after the stratification of patients by the level of anti-TF IgM and SNA binding we were able to select a group of cancer patients (29%) where the diagnostic accuracy reached 100%. It is important that the high accuracy of diagnostics in patients with a high SNA binding and low TF-IgM level was not appreciably dependent on the stage of disease or tumor morphology, indicating that this biomarker is highly suitable for early gastric cancer diagnostics. Our preliminary data in breast cancer show similar anti-TF Ab sialylation changes (unpublished). Notably, the similar stratification of patients and controls by SNA binding and anti-TF IgA level as has been done for SNA and IgM, showed that patients with high SNA binding and low IgA level demonstrated the ACC value equal to 52%, which allowed no cancer-noncancer group discrimination, thus supporting the idea that IgM is the main target for changes in the increased anti-TF antibody sialylation in cancer. However, the group is rather small. Therefore we consider these data as preliminary ones and a further pertinent study will be required to draw to any final conclusions.

The better survival of patients with a high level of anti-TF IgG antibodies supports our previous findings [12], and a similar association was also found for anti-TF IgM antibodies. No relation to survival was found for the SNA reactivity of TF-specific antibodies. However, a significant (negative) association of the SNA/anti-TF IgM index with survival was demonstrated that is a benefit in survival of patients with a low level of SNA/IgM index (Figure 6(c)) especially for patients with intestinal type tumors (Figure 6(d)). In contrast, the SNA/IgG and SNA/IgA indexes did not show such association.

Since the sialylated Abs display immunosuppressive or tolerogenic effects [40, 41], the higher sialylation of TF antigen-specific IgM Abs may have a negative effect on tumor immunity possibly by interfering with binding of more active anti-TF IgG to tumor cells. Alternatively, highly sialylated (anti-inflammatory) Abs may eliminate undesirable Ab-induced inflammatory reactions in tumor tissue that may promote tumor growth [42–44]. It is logical to assume that different immune mechanisms may be involved in Ab-mediated reactions with circulating tumor cells or tumor cells in tumor tissue, depending on the beneficial or detrimental microenvironment* in situ*. The functional activities of aberrantly glycosylated TF-specific antibodies remain to be determined. Our research currently aims to define the function of differently sialylated TF-specific Ab subsets and their further characterization.

Our findings support the idea that it is hardly possible to have a universal diagnostic biomarker applicable for all cancer patients even within a specific cancer site. This implies that, like cancer therapy, diagnostics also badly needs personalization and the success may be anticipated only if a proper test in a suitable subgroup of patients is applied. Thus the challenge is which criteria should be used to stratify correctly both patients and markers. Being present in each individual, naturally occurring anti-TF autoantibodies represent a good target for Abs glycoprofiling investigation in cancer detection, screening programs or risk factor studies, in contrast to many other tumor-related Abs that may be revealed in a minority of patients.

In conclusion, our findings are the first evidence that the sialylation of naturally occurring autoantibodies to the tumor-associated Thomsen-Friedenreich antigen is significantly increased in patients with gastric cancer. This increase is mainly related to the anti-TF IgM and IgA isotypes observed already at the early stages of cancer and is independent of tumor morphology or gender. Coming of the opposite character of changes in anti-TF IgM level and the sialylation degree of TF-targeting antibodies in cancer, a combination of these two parameters may be recommended as a novel biomarker for an early diagnosis of gastric cancer and disease prognosis. Such noninvasive approach may be a good prerequisite for the improvement of the clinical utility of antibody-based biomarkers. This information can be exploited for the structural-based functional study of antibodies to tumor-related glycans to further evaluate the clinical relevance of tumor-specific antibody glycovariants.

## Figures and Tables

**Table 1:** Characteristics of the subjects tested.

Group	*n*	Males	Females	M/F	Median age (range), yr
Donors	**31**	13	18	0.72	53.6 (31–70)
Benign diseases group^1^	**30**	16	14	1.14	65.0 (44–76)
Noncancer^2^	**61**	29	32	0.91	59.5 (31–76)
Cancer patients Stages 1–4	**142**	82	60	1.36	69.2 (25–84)
Stage 1	**39**	19	20	0.95	66.0 (28–84)
Stage 2	**32**	18	14	1.28	66.5 (46–80)
Stage 3	**51**	30	21	1.42	67.0 (37–76)
Stage 4	**20**	15	5	3.0	65.0 (49–81)

^1^A group of patients with chronic gastric diseases: peptic ulcer disease, *n* = 9; chronic gastritis, *n* = 11; and atrophic gastritis, *n* = 10. ^2^A combined group of donors and patients with nonmalignant stomach diseases. The number of subjects examined using a particular method is given in the corresponding section of the results.

**Table 2:** The sensitivity, specificity, and accuracy of diagnostics (ACC) for the main parameters under study.

	Sensitivity (95% CI)	Specificity (95% CI)	ROC curve area (95% CI)	*P* Value	ACC
Anti-TF IgA	0.68 (0.51–0.82)	0.77 (0.56–0.91)	0.70 (0.57–0.83)	0.007	0.719
Anti-TF IgG	0.55 (0.44–0.67)	0.58 (0.41–0.74)	0.53 (80.42–0.65)	0.543	0.563
Anti-TF IgM	0.66 (0.57–0.75)	0.69 (0.56–0.81)	0.69 (0.61–0.77)	<0.0001	0.670
SNA	0.60 (0.51–0.69)	0.62 (0.49–0.74)	0.66 (80.58–0.7)	0.000	0.611
SNA/anti-TF IgA	0.71 (0.54–0.85)	0.73 (0.52–0.88)	0.79 (0.67–0.91)	<0.0001	0.719
SNA/anti-TF IgG	0.66 (0.54–0.77)	0.60 (0.43–0.76)	0.64 (0.53–0.75)	0.017	0.643
SNA/anti-TF IgM	0.70 (0.61–0.78)	0.67 (0.54–0.79)	0.73 (0.66–0.80)	<0.0001	0.692

95% CI: 95% confidence interval (in brackets).

ACC: accuracy of diagnostics for each subgroup calculated by the ROC analysis as compared with that of the corresponding subgroup of controls (31 blood donors and 30 patients with nonmalignant gastric diseases). ACC = (TP + TN)/(TP + TN + FP + FN) where TP: true positive cases, TN: true negative cases, FP: false positive cases, and FN: false negative cases.

**Table 3:** Distribution of cancer patients by stage of disease, tumor morphology, and gender in subgroups stratified by TF-specific antibody reactivity to SNA and anti-TF IgM level.

Group of patients	*n*	SNA/IgM subgroup			
		1	2	3	4
		SNA≥IgM≥	SNA<IgM≥	SNA≥IgM<	SNA<IgM<
	124∗	25	37	36	26

Stage 1	43	8 (18.6)	14 (32.2)	13 (30.2)	8 (18.6)
Stage 2	28	7 (25.0)	7 (25.0)	8 (28.6)	6 (21.4)
Stage 3	39	7 (17.9)	13 (33.3)	12 (30.7)	7 (17.9)
Stage 4	14	3 (21.4)	3 (21.4)	3 (21.4)	5 (35.7)

DT	49	11 (22.4)	16 (32.7)	14 (28.6)	8 (16.3)
IT	73	14 (19.2)	19 (26.0)	22 (30.1)	18 (24.6)

males	70	15 (21.7)	18 (25.7)	20 (28.6)	17 (24.3)
females	54	10 (18.5)	19 (35.2)	16 (29.6)	9 (16.7)

*Two patients had morphologically mixed (unclassified) tumors.

In brackets: the percentage in the corresponding group.
